# Systematic Decision-Making for Using Technological Strategies to Implement Evidence-Based Interventions: An Illustrated Case Study

**DOI:** 10.3389/fpsyt.2021.640240

**Published:** 2021-05-14

**Authors:** Bo Kim, Sarah M. Wilson, Tiffany M. Mosher, Jessica Y. Breland

**Affiliations:** ^1^Center for Healthcare Organization and Implementation Research, VA Boston Healthcare System, Boston, MA, United States; ^2^Harvard Medical School, Boston, MA, United States; ^3^Center of Innovation for Accelerating Discovery and Practice Transformation, Durham VA Health Care System, Durham, NC, United States; ^4^Duke University School of Medicine, Durham, NC, United States; ^5^Center for Innovation to Implementation, VA Palo Alto Health Care System, Menlo Park, CA, United States

**Keywords:** implementation strategies, technology, effort vs. impact, behavioral health, evidence-based interventions

## Abstract

Technology can improve implementation strategies' efficiency, simplifying progress tracking and removing distance-related barriers. However, incorporating technology is meaningful only if the resulting strategy is usable and useful. Hence, we must systematically assess technological strategies' usability and usefulness before employing them. Our objective was therefore to adapt the effort-vs-impact assessment (commonly used in systems science and operations planning) to decision-making for technological implementation strategies. The approach includes three components – assessing the effort needed to make a technological implementation strategy usable, assessing its impact (i.e., usefulness regarding performance/efficiency/quality), and deciding whether/how to use it. The approach generates a two-by-two effort-vs-impact chart that categorizes the strategy by effort (little/much) and impact (small/large), which serves as a guide for deciding whether/how to use the strategy. We provide a case study of applying this approach to design a package of technological strategies for implementing a 5 A's tobacco cessation intervention at a Federally Qualified Health Center. The effort-vs-impact chart guides stakeholder-involved decision-making around considered technologies. Specification of less technological alternatives helps tailor each technological strategy within the package (minimizing the effort needed to make the strategy usable while maximizing its usefulness), aligning to organizational priorities and clinical tasks. Our three-component approach enables methodical and documentable assessments of whether/how to use a technological implementation strategy, building on stakeholder-involved perceptions of its usability and usefulness. As technology advances, results of effort-vs-impact assessments will likely also change. Thus, even for a single technological implementation strategy, the three-component approach can be repeatedly applied to guide implementation in dynamic contexts.

## Introduction

Technology shapes both interventions and strategies for implementing interventions. Technological interventions are receiving increased attention and undergoing enhanced specification [e.g., (1)]. For mental healthcare, behavioral intervention technologies are being actively specified and evaluated for their potential to both broaden the interventions' reach and deliver the interventions through previously unexplored modalities (2, 3). However, such specification is not yet available for technological implementation strategies, including when and how to use them.

We define technological implementation strategies as “methods or techniques that use information and communications technology to enhance the adoption, implementation, and sustainability of a clinical program or practice.” This definition builds on the World Health Organization's definition of eHealth (“the use of information and communications technology in support of health and health-related fields”) (4) and Proctor and colleagues' definition of implementation strategies (“methods or techniques used to enhance the adoption, implementation, and sustainability of a clinical program or practice”) (5). Specifically, our definition is a combination of WHO's definition (which pertains to technology as it is used in health and health-related fields, without mention of implementation strategies) and Proctor and colleagues' definition (which pertains to implementation strategies, without mention of technology). As our work discusses technological implementation strategies that are at the cross-section of the two concepts, our definition pulls together the two pre-existing definitions.

Technology can improve implementation strategies' efficiency, simplifying progress tracking and removing distance-related barriers. For example, virtual implementation facilitation that uses telecommunication to implement measurement-based mental health care in the primary care setting (6) minimizes the need for implementation experts' in-person travel to the setting. Electronic audit-and-feedback, as opposed to feedback delivered verbally or by paper, can speed the implementation of treatment guidelines for concurrent substance use and mental disorders (7).

As technology advances, and as the implementation science field seeks innovative implementation strategies, there are increasing opportunities for new technological implementation strategies. But incorporating technology is meaningful only if the resulting strategy is both usable (i.e., is easy to use) and useful (i.e., helps improve performance/efficiency/quality – e.g., by supporting equitable access to healthcare services for vulnerable populations). Hence, we must systematically assess technological strategies' usability and usefulness before employing them. This assessment is much needed for implementing evidence-based mental healthcare interventions, given that the aforementioned examples of strategies like virtual implementation facilitation and electronic audit-and-feedback are playing an increased role in implementation. The growing number of options for technological implementation strategies calls for an approach (such as ours to be introduced in this article) that implementation efforts can use to methodically decide whether and how to use the strategies.

In this article, we (i) outline an adaptation of the effort-vs-impact assessment method from systems science / operations planning (8) as a three-component approach to this decision-making and (ii) to illustrate the approach, use as a case study the development of a package of implementation strategies for increasing evidence-based tobacco cessation at a Federally Qualified Health Center (FQHC). Namely, section The Three-Component Approach to Decide Whether/How to Use a Technological Implementation Strategy describes the general three-component approach, section Case Study: Developing a Package of Technological Implementation Strategies to Promote Evidence-Based Tobacco Cessation at an FQHC describes a specific application of the approach, and section Discussion summarizes the approach and discusses its implications.

## The Three-Component Approach to Decide Whether/How to Use a Technological Implementation Strategy

**Figure 1:**
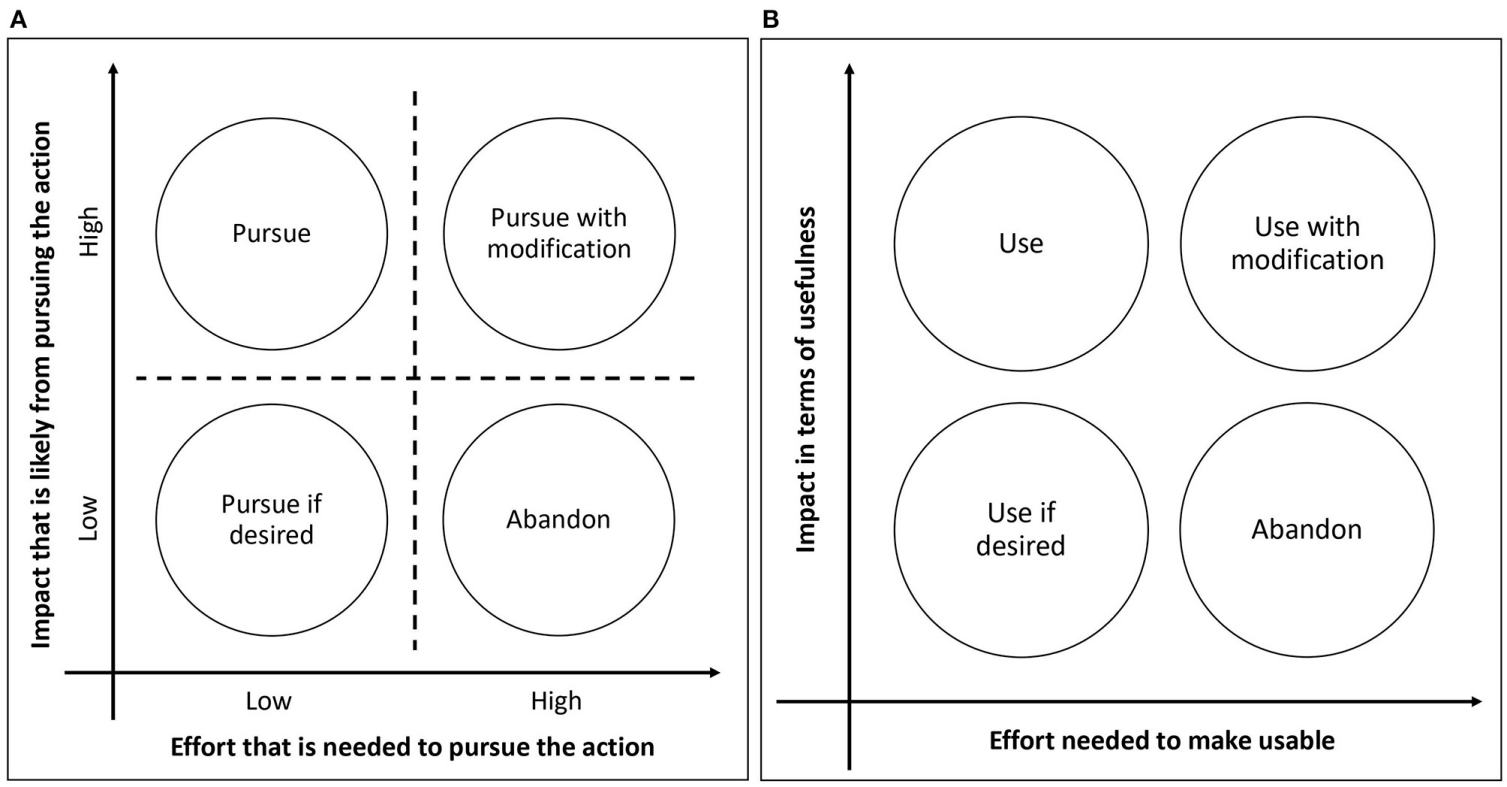
**(A)** An effort-vs-impact chart. **(B)** An adapted effort-vs-impact chart for assessing whether/how to use a technological implementation strategy.

The three components are – an assessment of the effort required to use the strategy, an assessment of the strategy's potential impact, and a decision whether/how to use the strategy. The approach uses a chart-based visualization (Figure 1A) to categorize a potential action as (i) requiring little effort to make a big impact, (ii) requiring little effort to make a small impact, (iii) requiring a lot of effort to make a big impact, and (iv) requiring a lot of effort to make a small impact. The combined consideration of the effort (low/high) and the impact (low/high) serves as a rubric for deciding whether/how to pursue the action [in our case, “action” refers to the use of a technological implementation strategy – e.g., using electronic audit-and-feedback to implement treatment guidelines for concurrent substance use and mental disorders (7)]. For the electronic audit-and-feedback example, effort considerations could involve the potential time and resources needed to install and train mental healthcare staff in the electronic audit-and-feedback software, and impact considerations could involve the potential increase in mental healthcare staff's awareness of implementation progress, in turn encouraging higher use of the evidence-based intervention.

The method is widely applied to operations planning (9, 10), and increasingly used in healthcare operations and quality improvement [e.g., the Agency for Healthcare Research and Quality (AHRQ)'s guide to implementing a pressure injury prevention program (11) recommends the method for prioritizing among interventions.] For example, Kashani and colleagues used the effort-vs-impact assessment as a part of implementing quality improvement initiatives that involved critical care fellows (12), and Fieldston and colleagues used the assessment as a key tool for rapid-cycle improvements that were implemented at a large independent hospital (13). These are just a few examples, and the effort-vs-impact assessment has been used by numerous initiatives, from individual clinics to health care systems, to prioritize and select among potential interventions to implement change in health care delivery (14–16). For each intervention, the needed effort for implementation (e.g., whether needed resources are already available) and the likely impact from implementation (especially compared to other options) are considered. The utility of the approach is underpinned by strategic change management concepts (17), and we describe below how we adapted the method for decision-making around technological implementation strategies.

### Before Initiating the Three-Component Approach

As for any implementation strategy, the technological implementation strategy of interest must be named, defined, and specified (5) prior to initiating the three-component approach, to ensure that all stakeholders have an explicit, shared understanding of the strategy. For instance, a description of a strategy that adds clinical reminders [e.g., on compliance with a mental health clinical practice guideline (18)] should specify (i) what triggers the reminder, (ii) how often, (iii) for whom, and (iv) which actions qualify as attending to the reminder. Aligning to an established framework in describing the strategy [e.g., (5)] is recommended, as this facilitates subsequent comparisons to other strategies (both technological and non-technological).

### Throughout the Approach

Comprehensive stakeholder involvement and consideration of alternatives are key. Each component should directly involve potential users of the technological implementation strategy to gauge familiarity with the technology. Also important is continuous input from the health system's technology infrastructure leads and decision makers (e.g., information technology department), to gauge the availability of resources for any infrastructure changes and trainings the technology would require. For example, trainings for using virtual communication platforms are a key aspect of implementing the delivery of evidence-based telemental healthcare (particularly increased in prevalence due to COVID-19) (19). Explicit articulation of potential alternative implementation strategies is recommended, so that the strategy being considered can be compared alongside expected levels of effort and impact of using alternatives.

### Component 1: Assess the Potential Effort Needed to Make the Technological Implementation Strategy Usable

This first component aims to answer:

- How available is the technological infrastructure (e.g., equipment, information system) to potential users (i.e., individuals or teams responsible for conducting the implementation)?
- How familiar is the technology to potential users – e.g., how much training is needed?

A variety of methods can be used to gather and analyze stakeholder input on these questions. Choice of methods should be based on feasibility and can include questionnaires, facilitated group discussion, or both. The resulting assessed potential effort (e.g., expected effort to use a virtual communication platform for implementing evidence-based telemental healthcare) could thus be expressed quantitatively [e.g., if the questionnaire asked for responses on a Likert scale (20, 21)] or qualitatively [e.g., regarding consensus reached during the discussion (22, 23)]. Regardless of the method of assessing effort, defining “more or less effort compared to what” is necessary to ensure that stakeholders provide constructive feedback. Hence, as mentioned above, it is important to explicitly articulate and share available alternative implementation strategies.

### Component 2: Assess the Potential Impact (i.e., Usefulness) of Using the Technological Implementation Strategy

This second component aligns with Shaw and colleagues' three eHealth domains (24) and aims to answer: To what extent would the technological strategy enable

- better monitoring, tracking, and informing of implementation progress?
- better communication among implementation stakeholders?
- better collection, management, and use of implementation data?

To ensure that innovative technologies do not inadvertently increase health disparities, this component also assesses to what extent the technological strategy might increase, maintain, or reduce disparities.

Notably, this component need not be conducted separately from Component 1 – e.g., the same questionnaire and/or group discussion can be used to gather stakeholder perspectives, or Component 2 can come before Component 1. Similar to the assessed effort under Component 1, the resulting assessed potential impact can be expressed quantitatively or qualitatively, and articulation of alternative strategies for comparison is highly recommended. Importantly, conducting these components does not preclude implementation teams from using a separate conceptual model or framework to guide their planning and/or evaluation (25). Rather, such a model or framework can provide a systematic structure through which to assess potential effort and impact as outlined under Components 1 and 2. For example, mental health-related implementation efforts being guided by the Integrated Promoting Action on Research Implementation in Health Services model (26) – e.g., (27) – can specifically assess whether the technological strategy would enable better communication between the facilitators and the recipients of the implementation, as defined by the model's “Facilitation” and “Recipients” constructs, respectively.

### Component 3: Decide Whether to Use or Abandon the Technological Implementation Strategy

This component, based on the potential effort (i.e., usability) and potential impact (i.e., usefulness) assessed through Components 1 and 2 above, places the technological implementation strategy being considered onto the effort-vs-impact chart, now adapted to the context of deciding whether to use the strategy for implementation (Figure 1B). For instance, when considering the use of electronic audit-and-feedback to implement mental health treatment guidelines (7), potential effort might include needed trainings to learn the electronic audit-and-feedback system, while potential impact might include increased staff awareness of implementation progress (and consequently increased motivation to work on implementation). Especially when it is unclear which quadrant the strategy belongs to, it can help to place the previously articulated alternative strategies on to the same chart for comparison. As is the case for Components 1 and 2, it is recommended that this component closely involve stakeholders (28), who can clarify their previously expressed perspectives, when needed, to collaboratively reach a decision with the implementation team.

## Case Study: Developing a Package of Technological Implementation Strategies to Promote Evidence-Based Tobacco Cessation at an FQHC

We present an ongoing implementation pilot study that illustrates our approach for decision-making around technological implementation strategies. This study, entitled *Stakeholder-Engaged Implementation of Smoking Cessation Health Services* (PI: SW), is developing a tailored package of technological implementation strategies to facilitate adoption and sustainment of an evidence-based tobacco cessation intervention [the 5 A's (29)] at an FQHC. Potential barriers to integration of technological tools for tobacco cessation include disruption of clinic workflow, belief that technology is burdensome, and perceived lack of usefulness of technology (30, 31). Since technology use can be affected by multiple contextual factors (32), it is essential for implementation to account for these factors. Therefore, the study combined implementation mapping methodology (33) with our three-component approach to choose implementation strategies.

### Implementation Setting and Population

The implementation site is an FQHC where 75% of patients are at or below 200% of the federal poverty level (34). Intervening on patient tobacco use was deemed crucial by the organization because tobacco use remains a leading cause of death in their region (35) and is highest among those at or below 200% of the federal poverty level (36). The FQHC serves over 30,000 patients per year, 92% of whom identify as a racial and/or ethnic minority (49% Latinx and 73% Black/African American) and 55% of whom are uninsured. Patients have a variety of healthcare needs including tobacco/vaping cessation, substance abuse treatment, preventative care, urgent care, acute illness, and management of chronic illnesses. Clinic staff at the FQHC have diverse educational backgrounds, clinical roles, and specializations (adult vs. pediatric). Staff include physicians, physician assistants, nurse practitioners, nursing staff, medical assistants, and behavioral health specialists. At the FQHC, previous efforts to implement an evidence-based specialty smoking cessation clinic were not sustained due to staff turnover. The interdisciplinary research team, led by SW, developed an innovative implementation plan to increase provision of evidence-based tobacco cessation at the FQHC. Research staff entered a formal collaborative arrangement with FQHC clinical and administrative leadership in terms of sharing research project decision-making.

### Target Intervention

The purpose of this project was to improve adherence to the target intervention, the “5 A's.” The 5 A's intervention is part of the AHRQ clinical guidelines for tobacco cessation (29). It consists of five sequential steps to tobacco cessation: Ask, Advise, Assess, Assist, and Arrange. Despite evidence supporting its effectiveness, medical provider adherence rates to the 5 A's are low (37). Moreover, there are documented healthcare inequities nationally in the provision of tobacco cessation treatment (38). Specific to the Assist and Arrange steps at this FQHC, previous efforts to implement an evidence-based specialty tobacco cessation clinic encountered barriers to adoption, and ultimately the tobacco cessation clinic was not sustained due to staff turnover. Additionally, for pediatric healthcare providers, there are key barriers to completing the Ask step regarding electronic nicotine delivery systems (ENDS; e.g., e-cigarettes or vape pens), including inadequate screening tools and providers lacking information on slang language (39).

### Overall Study Design and Participants: Applying the Three-Component Approach

All study procedures were approved by the Duke University Health System Institutional Review Board. The study used implementation mapping methodology (33) combined with the three-component approach outlined above. First, the study team created a logic model of the problem (i.e., outline of barriers to 5 A's completion). A sample of *N* = 12 healthcare staff members were recruited to complete telephone interviews. Quota sampling was used to ensure representation of different staff types and clinic types, to adequately assess the potential clinic-specific barriers to 5 A's completion. Staff participants included two medical assistants, one patient educator, one nurse, two behavioral health specialists, two advanced practice providers, and four physicians. Participants worked in varying contexts, including Pediatrics, Family Medicine, and Internal Medicine. See below for qualitative methodology and findings from each component. Interviews were audio recorded and transcribed. Transcriptions were then analyzed using RADaR technique (40) to detect salient themes in the data.

Following interviews with staff, we developed a logic model of change through a guided survey with FQHC administrators and clinic leads (*N* = 7). Leaders (e.g., medical chiefs, supervisors, and administrators) were recruited who oversaw varying types of clinics and staff, including Behavioral Health, Pediatrics, Family Medicine, and Internal Medicine. A combination of close-ended numeric questions and open-ended text questions were asked. Quantitative questions were analyzed using exploratory descriptive statistics. Open-ended survey responses were analyzed using RADaR technique (40).

Full results of the study are reported elsewhere (41). For the purpose of this methodology paper, we will focus on one specific potential implementation strategy. In creating the Logic Model of the Problem, one theme of staff interviews was an emphasis on numerous priorities and tasks during clinical encounters with patients. This long task list for clinical encounters was contrasted with often having insufficient time. Completing the 5 A's was viewed as important but difficult because it can be seen as “one more thing to do” and may be de-prioritized over other seemingly more urgent clinical matters.

In creating the Logic Model of Change (i.e., next step toward creating an implementation package), we ascertained that integrating each step of the 5 A's into the electronic health record (EHR) could help overcome time and effort barriers to 5 A's adherence, since it would both remind staff to complete tobacco screenings as well as clearly communicate that assessing and treating tobacco use is a high programmatic priority. Below we detail how the three-component approach was used to assess the potential effort and impact of integrating the 5 A's into the EHR (Note: “Effort” and “impact” mentioned in subsections Component 1: Assess the Potential Effort Needed to Make the “Integrating the 5 A's Into the EHR” Strategy Usable through Component 3: Assess the Potential Effort-vs-Impact of the “Integrating the 5 A's Into the EHR” Strategy to Decide Whether to Use, Use-if-Desired, Use-With-Modification, or Abandon It refer to “expected effort” and “expected impact.”).

### Component 1: Assess the Potential Effort Needed to Make the “Integrating the 5 A's Into the EHR” Strategy Usable

#### Procedure

Within interviews with staff participants, study staff asked the following questions related to the potential effort of integrating the 5 A's into the EHR: What are your thoughts about our embedding the 5 A's into [EHR Name]? What might be specific barriers? What are possible issues or complications that may arise in using a computerized 5 A's tool in [EHR Name] in your clinic? To what extent might the implementation of a computerized 5 A's tool in [EHR Name] compete with other workload demands and patient care priorities in your clinic?

Building on the staff interview findings, the guided survey for FQHC administrators and clinic leaders asked, for each step of the 5 A's, “How much logistical/organizational effort will it take to integrate the [Ask/Advise/Assess/Assist/Arrange Step] into [EHR Name]?” Leaders were asked to consider how compatible the tool would be with current clinic flow as well as how much effort it would take to train staff to use the new tool. Responses were entered on a 0 to 10 point scale, where 0 indicated “no effort at all” and 10 indicated “maximum effort.” Questions were also asked regarding how to ensure that each 5 A's step is consistently completed, and what relevant performance objectives need to be established.

#### Results

Data from the staff interviews underwent the RADaR technique (40)'s structured data charting and data reduction steps to arrive at the following themes related to the potential effort of integrating the 5 A's into the EHR. (i) The most salient effort-related theme (voiced by *n* = 7 participants) was concern that adding items to the EHR (which already contains many features and forms) could be potentially burdensome to staff (Wilson et al., (41) in preparation). (ii) However, other staff members downplayed the effort of integrating the 5 A's (*n* = 4), with one participant noting that integration is less burdensome “if done the right way.”

See Table 1 for a summary of findings from the FQHC leaders' survey. There was variability across steps of the 5 A's with regard to how much effort would be involved in integrating that step into the EHR. Applying the RADaR technique (40) to the open-ended survey data gave rise to the following themes related to considerations for effort. (i) Some steps of the 5 A's (most notably the first “Ask” step) were already being captured in the EHR. (ii) Also, ancillary tasks and features would require additional effort (e.g., setting up trainings, peer reviews of documentation, and tracking dashboards).

**Table 1:** Leaders' perceived potential effort and impact of integrating the 5 A's into the EHR.

		**Ask step**	**Advise step**	**Assess step**	**Assist step**	**Arrange step**
Perceived potential effort	Range	1–5	2–7	0–7	2–9	2–10
	M (SD)	2.3 (1.4)	5.9 (1.7)	2.4 (2.2)	4.9 (2.3)	5.4 (3.3)
Perceived potential impact	Range	1–10	3–9	1–10	4–10	6–10
	M (SD)	6.7 (2.9)	6.7 (2.0)	6.6 (3.0)	7.3 (2.1)	8.1 (1.6)
**Decisional quadrant**						
Low effort/high impact (Proceed)		71%	29%	71%	29%	43%
High effort/high impact (Proceed with modifications)		14%	57%	14%	57%	57%
Low effort/low impact (Proceed if desired)		14%	0%	14%	14%	0%
High effort/low impact (Abandon)		0%	14%	0%	0%	0%

*Columns may not add up to 100% due to rounding*.

### Component 2: Assess the Potential Impact (i.e., Usefulness) of Using the “Integrating the 5 A's Into the EHR” Strategy

#### Procedure

Clinical staff participants were asked several questions related to the potential impact of integrating the 5 A's into the EHR: What are your thoughts about our embedding the 5 A's into [EHR Name]? What would be helpful about doing this? Will it replace or enrich current stop-smoking practices? Why/how?

Interviews, focus group discussions, and structured questionnaires are assessing the impact of using the “Integrating the 5 A's into the EHR” strategy. The research team is seeking additional participant perspectives regarding the benefits of integrating the 5 A's for tobacco cessation into the EHR, including how it may enrich current tobacco cessation practices. Then, building on the interviews, the guided survey for clinic leaders asks, for each step of the 5 A's, how the tool will help enhance the quality, tracking/monitoring, and communication around the clinic's tobacco cessation care.

#### Results

Data from the staff interviews were methodically charted and structurally reduced using the RADaR technique (40), which led to the following themes related to the potential organizational and clinical impact of integrating the 5 A's into the EHR. (i) A majority of staff participants (*n* = 7) noted the helpfulness of integrating the 5 A's more closely into the EHR. (ii) One participant did not see the 5 A's as helpful to their patient population, noting that they did not view the prevalence of tobacco use in their pediatric population as an issue [Regarding this perspective, it is important to note that – contrary to this stated staff opinion – pediatric clinical guidelines recommend using the 5 A's for tobacco/vaping use for every adolescent patient (42)]. (iii) Regarding clinical impact, staff participants also noted several logistical barriers to 5 A's fidelity that would not be overcome merely by integrating the 5 A's into the EHR, such as billing not covering follow-up calls, overbooking in clinics, and staff burnout.

Regarding organizational leadership perspectives on the impact of integrating the 5 A's into the EHR, assessments of impact varied (Table 1). In general, perceived potential impact was rated highly. Open-ended responses were analyzed using the RADaR technique (40), which indicated the following themes. (i) Leaders had varying perspectives on how much impact would be made by integrating the 5 A's into the EHR because some elements of the 5 A's were already captured within the EHR. (ii) Additionally, there were several ideas from leadership regarding ways to maximize impact (e.g., supervisor dashboards, peer review of charted notes, requiring “hard stops” that would not allow a provider to complete an encounter until tobacco use is assessed).

### Component 3: Assess the Potential Effort-vs-Impact of the “Integrating the 5 A's Into the EHR” Strategy to Decide Whether to Use, Use-if-Desired, Use-With-Modification, or Abandon It

#### Procedure

In the leader survey, a smart design was used to route leader participants to different questions based on their effort vs. impact ratings. Each 0 to 10 rating was categorized as “low” (less than 5) or “high” (5 or greater). Based on these categorizations for each 5 A's step, responses were categorized as: (1) high impact/high effort – use with modifications, (2) high impact/low effort – use, (3) low impact/high effort – abandon, or (4) low impact/low effort – use if desired. Leaders were then presented with an image and text feedback tailored to their responses. This tailored feedback stated which effort-vs-impact decisional quadrant they fell into, and what the typical way forward for that quadrant would be (e.g., use with modification). Each leader was asked open-ended questions about whether they agreed and what next steps would be involved.

#### Results

From the leader survey, few responses fell into the low effort/low impact or high effort/low impact quadrants. For those who indicated these responses, concerns mainly centered around the fact that some aspects of the 5 A's were already captured in the EHR. However, from our staff participant interviews, it was clear that beyond the Ask and Assess steps, there were not streamlined processes in the EHR for guiding and documenting other steps of the 5 A's intervention.

### Summary of the Case Study

This case study demonstrates how our three-component approach is being used to methodically incorporate key stakeholder perspectives on specific contextual factors and potential barriers surrounding the technological implementation strategy (Integrating the 5 A's into the EHR) as it pertains to the 5 A's, to shape whether/how to use the strategy. The approach is particularly valuable to the research team and the stakeholders at the FQHC for aligning the implementation strategy to the FQHC's priorities and workflow. Use of the three-component approach facilitated concrete discussions with stakeholders regarding practicalities of using digital implementation strategies. It also helped situate this particular implementation strategy within the organizational priorities of the health center. This method also prompted stakeholders to identify additional implementation strategies that were digital (e.g., supervisor dashboard) and non-digital (e.g., a tailored clinic workflow plan, training integrated into the clinic workflow).

## Discussion

We provide a three-component approach to assessing whether/how to use a technological strategy for implementing an intervention. To present the approach in a broad-to-specific way, we first describe the general approach (section The Three-Component Approach to Decide Whether/How to Use a Technological Implementation Strategy). Then, we illustrate the approach (section Case Study: Developing a Package of Technological Implementation Strategies to Promote Evidence-Based Tobacco Cessation at an FQHC) using a case study of decision-making around technological implementation strategies to promote evidence-based tobacco cessation at an FQHC, specifically describing how the strategy of “Integrating the 5 A's into the EHR" was considered for implementing the cessation intervention targeted at a largely low-income, uninsured, and racial/ethnic minority population. The approach directly contributed to the case study implementation's objective to closely engage multiple stakeholders in devising a contextually appropriate package of technological implementation strategies.

The approach is an adaptation of the effort-vs-impact assessment method, which is widely used in healthcare quality improvement. The approach includes stakeholders throughout and identifies alternative (both technological and non-technological) strategies so that stakeholders can compare options, ultimately deciding on the strategy that provides the minimal effort to impact ratio. This approach systematically assesses technological implementation strategies, going beyond a framework that merely notes the domains by which technological strategies can be characterized (e.g., usability, usefulness).

Given our focus on providing a “how-to” approach rather than a framework (i.e., an approach for assessing whether/how to use a technological implementation strategy), implementation projects that already have a guiding framework can still benefit from using our approach. For instance, the case study described above is guided by the Consolidated Framework for Implementation Research (43), and it can thus align to the framework's “Available Resources” construct when assessing whether the necessary technology infrastructure and/or capacity for technology training are available (i.e., Component 1 of our three-component approach).

As noted above, whether assessing a strategy's potential effort or impact (i.e., Components 1 and 2) is done quantitatively or qualitatively depends on what is feasible for the implementation team (e.g., administering a questionnaire, holding a group discussion, or both). Even if the axes of the effort-vs-impact chart do not have quantitative units associated with them, the chart can serve as a helpful conversation tool for the implementation team and their stakeholders, when discussing the relative effort and impact of the strategy and its alternatives. The case study above does exactly this, where each leader's perceived effort-vs-impact of the “Integrating the 5 A's into the EHR” strategy is visualized on the chart to facilitate both confirmation of the leader's perceptions about the technological implementation strategy and brainstorming of next steps to feasibly incorporate the strategy into the implementation effort.

Technological strategies vary widely, from supporting a mainly human-operationalized strategy to enabling a fully technology-operationalized strategy. For example, participatory system dynamics uses human-informed implementation simulations that are computationally generated, which in turn inform human-driven implementation (44). And types of technological strategies will certainly change over time, potentially toward increased automation [e.g., avatars for technical assistance (45)]. For the case study above, for instance, further automated tracking/monitoring of tobacco cessation care may alter the extent to which stakeholders consider the “Integrating the 5 A's into the EHR” strategy to require more or less effort.

As such, as technology advances, results of effort-vs-impact assessments of strategies will likely change. For example, natural language processing is currently effortful and computationally expensive, but may not be in the future. Evolving views on privacy will also affect how and whether health systems can use passively captured data for health purposes. Thus, even for a single technological implementation strategy, our approach is meaningful to repeat when the strategy is considered under different location and temporal contexts. For instance, prior to the current COVID-19 pandemic, using phone/video to implement evidence-based treatments may have been considered higher effort and potentially lower impact than their in-person alternatives. However, with social distancing requirements (46) and suspension of relevant HIPAA rules around telecommunication (47), using phone/video may now be considered lower effort compared to the newly heightened effort of ensuring safety from viral transmission for in-person strategies.

There are limitations to this work. We present here a case study of the three-component approach's application, and the approach is yet to be applied and evaluated across multiple behavioral health interventions. Relatedly, the approach has not been used for implementing interventions across multiple target populations. In light of these limitations, a notable strength of this work is that the components of the approach are reliant neither on population nor content specifics of our case study example, which will enable the approach to be applicable to other behavioral health interventions. Another strength is that the work is grounded in the well-established effort-vs-impact assessment method, which has been successfully used both within and beyond the health care realm.

Further work is needed to examine the approach's applicability to implementation for different vulnerable populations and beyond behavioral health. Technology is both a potential countermeasure to health disparities (e.g., better enabling access to healthcare) and a potential threat that further excludes vulnerable populations (e.g., those without equitable access to innovative technologies) (48). Therefore, especially as health disparities are increasingly viewed as an important component of implementation research and practice (49, 50), an approach such as ours that systematically assesses the effort-vs-impact balance of technological implementation strategies is essential to helping ensure that technology is optimally used based on the context into which evidence-based interventions are implemented. Particularly when considering the variations in population-based needs and availability of resources for global mental health, our three-component approach can help methodically approach deciding whether a certain technological implementation strategy is appropriate for a certain mental healthcare setting.

## Data Availability Statement

The original contributions presented in the study are included in the article, further inquiries can be directed to the corresponding author.

## Ethics Statement

The studies involving human participants were reviewed and approved by Duke University Health System Institutional Review Board. The ethics committee waived the requirement of written informed consent for participation.

## Author Contributions

BK, SW, and JB were key conceivers of the ideas presented in this Perspective. BK led the writing of the manuscript. SW, TM, and JB provided critical revisions to the manuscript's intellectual content. All authors reviewed the final version of the manuscript.

## Conflict of Interest

The authors declare that the research was conducted in the absence of any commercial or financial relationships that could be construed as a potential conflict of interest.
